# Supplementary material for: The Usage and Trustworthiness of Various Health Information Sources in the United Arab Emirates: An Online National Cross-Sectional Survey
Source: Healthcare (Basel). 2023 Feb 24;11(5):663. doi: 10.3390/healthcare11050663 (PMC10001002; doi:10.3390/healthcare11050663)
Supplement: Supplementary file 1 [file healthcare-11-00663-s001.zip › healthcare-2199203-supplementary/Supplementary Material - Questionnaire.pdf]

Health Information Sources in the United Arab Emirates All changes saved in Drive

Send

Questions Responses Settings

Section 1 of 4

Health Information Sources in the United Arab Emirates

We are a group of medical students at the University of Sharjah conducting a research project about the "Health Information Sources in the United Arab Emirates." The purpose of this study is to evaluate the different health information sources used by the population in the United Arab Emirates and the level of trust associated with them.

You have been selected to participate in this study and your participation is strictly voluntary. If you agree to participate, you will be asked to fill out a questionnaire that should take 5 to 8 minutes of your time. There are no risks associated with participation in this study.

We assure you that your responses will be confidential and will be used only for research purposes. Hence, after submitting your response, it cannot be removed from the database as it is no longer identifiable. There will be no benefits gained from participation in this study.

If you have any questions regarding this study, please feel free to contact our Research Supervisor - Dr. Hiba Barqawi at hbarqawi@sharjah.ac.ae or 06-5057268.

For any further ethical concerns, you may contact the Research Ethics Committee at University of Sharjah at rec@sharjah.ac.ae – 06-5057304.

Thanks in advance for your participation.

لإجابة الإستبيان باللغة العربية اضغط هنا:
XXXXXXXXXXXXXXXXXXXXXXXXX

After section 1 Continue to next section

Section 2 of 4

Untitled section

Description (optional)

Please check the box below to proceed to the survey: *

- [ ] I have read the above information and agree to participate in this research.

Do you live in the UAE? *

- ○ Yes
- ○ No

After section 2 Continue to next section

Section 3 of 4

Demographics

Please choose the option that best describes you.

Sex: *

1. Male
2. Female

Age: *

Short answer text

Highest Degree Obtained: *

1. Middle School or lower
2. High School
3. Diploma/ Bachelor's Degree
4. Postgraduate Degree (MSc, PhD., etc.) or higher

Marital Status: *

1. Single
2. Married
3. Divorced
4. Widowed

Nationality: *

1. UAE National
2. Other Arab
3. Non-Arab

Place of Residence: *

1. Abu Dhabi
2. Dubai
3. Sharjah
4. Ajman
5. Umm Al Quwain
6. Ras Al Khaimah
7. Fujairah

Field of work: *

1. Healthcare (Nurses, Doctors, Dentists, Pharmacists, Healthcare Administration, etc.)
2. Non-Healthcare
3. Student (Health Sciences, Medicine, Dentistry, etc.)
4. Student (Other non-health related majors)
5. Housewife
6. Unemployed (healthcare background)
7. Unemployed (non-healthcare background)

Do you have health insurance? *

1. Yes
2. No

How would you rate your health? *

	1	2	3	4	5	
Poor	○	○	○	○	○	Excellent

How often do you need to have someone help you when you read instructions, pamphlets, or other written material from your doctor or pharmacy? *

1. Never
2. Rarely
3. Sometimes
4. Often
5. Always

Do you have any long-term medical conditions (such as High Blood Sugar, High Blood pressure, Liver or Kidney diseases, etc.) *

1. Yes

2. No

After section 3 Continue to next section

Section 4 of 4

Untitled Section

Description (optional)

In the last 12 months (During COVID-19), which of the following health information sources have you used (Choose all that apply): *

A health information source is any resource that you use to learn more about health, illnesses, medications, etc.

- [ ] Doctor
- [ ] Internet (Social Media)
- [ ] Internet (Websites and Blogs)
- [ ] Friends and Family
- [ ] Printed Materials (Brochures, Books, Newspapers, Articles, ...)
- [ ] Pharmacists
- [ ] Other...

Before the COVID-19 pandemic (Before 2020), which of the following health information sources have you used (Choose all that apply): *

A health information source is any resource that you use to learn more about health, illnesses, medications, etc.

- [ ] Doctor
- [ ] Internet (Social Media)
- [ ] Internet (Websites and Blogs)
- [ ] Friends and Family
- [ ] Printed Materials (Brochures, Books, Newspapers, Articles, ...)
- [ ] Pharmacists
- [ ] Other...

Out of the previous sources, which would be your MAIN source for health information (Choose only one): *

- ○ Doctor
- ○ Internet (Social Media)
- ○ Internet (Websites and Blogs)
- ○ Friends and Family
- ○ Printed Materials (Brochures, Books, Newspapers, Articles, ...)
- ○ Pharmacists
- ○ Other...

How often do you use the internet as a health information source? *

	1	2	3	4	5	
Never	○	○	○	○	○	Always

Which of the following do you use the internet for? *

- [ ] Learn more about symptoms, diseases or medical conditions.
- [ ] Self-manage health or health conditions.
- [ ] Decide if a visit to the doctor is needed.
- [ ] [illegible]

- [ ] Choose a healthcare provider.
- [ ] Find more information after a doctor visit.
- [ ] Exploring treatment options
- [ ] Modify health and lifestyle behaviors.
- [ ] Looking up COVID-19 related information.
- [ ] Other...

Which of the following websites do you regularly use? *

- [ ] Local government websites (DHA, DOH, MOHAP, ...)
- [ ] International Health Bodies (WHO, CDC, NHS, ...)
- [ ] Wikipedia
- [ ] Search engines (Google, ...)
- [ ] Electronic newspapers (Khaleej Times, Gulf Times, UAEBarq, 3meed_news, ...)
- [ ] Health Blogs
- [ ] Other...

How much do you trust each of the following sources?

Description (optional)

Doctor *

	1	2	3	4	5	6	
Not at all trustworthy	○	○	○	○	○	○	Very trustworthy

Social Media *

	1	2	3	4	5	6	
Not at all trustworthy	○	○	○	○	○	○	Very trustworthy

Internet *

	1	2	3	4	5	6	
Not at all trustworthy	○	○	○	○	○	○	Very trustworthy

Friends and Family *

	1	2	3	4	5	6	
Not at all trustworthy	○	○	○	○	○	○	Very trustworthy

Printed Materials (Brochures, Books, Newspapers, Articles, ...) *

	1	2	3	4	5	6	
Not at all trustworthy	○	○	○	○	○	○	Very trustworthy

Pharmacists *

	1	2	3	4	5	6	
Not at all trustworthy	○	○	○	○	○	○	Very trustworthy
