# Supplementary material for: The Usage and Trustworthiness of Various Health Information Sources in the United Arab Emirates: An Online National Cross-Sectional Survey
Source: Healthcare (Basel). 2023 Feb 24;11(5):663. doi: 10.3390/healthcare11050663 (PMC10001002; doi:10.3390/healthcare11050663)
Supplement: Supplementary file 1 [file healthcare-11-00663-s001.zip › healthcare-2199203-supplementary/Supplementary Tables S1,2 and 3 .pdf]

Doctor Trustworthiness – Binary Logistic Regression (LR)						
**Model Terms**		**e^{β_i}**	**95% CI**	**SE**	**z-Statistic**	**P value**
**Intercept (β_0)**		**4.387**	**2.147 - 8.962**	**0.365**	**4.056**	**<0.0005**
**Comorbidities**	No	-	-	-	-	-
	Yes	0.689	0.455 - 1.044	0.212	-1.757	0.079
**Age**	Younger than or equal to 18 years	-	-	-	-	-
	Between 19 and 29 years, inclusive	1.120	0.629 - 1.994	0.294	0.386	0.699
	Between 30 and 39 years, inclusive	1.479	0.636 - 3.442	0.431	0.909	0.364
	40 years or older	1.421	0.613 - 3.297	0.429	0.818	0.413
**Marital Status**	Unmarried	-	-	-	-	-
	**Married**	**0.450**	**0.248 - 0.820**	**0.305**	**-2.613**	**0.009**
**Occupation**	Healthcare (nurses, doctors, dentists, pharmacists, healthcare administration, etc.)	-	-	-	-	-
	Housewife	1.306	0.647 - 2.635	0.358	0.746	0.456
	Non-healthcare	1.363	0.781 - 2.377	0.284	1.090	0.276
	**Student - health-related majors**	**1.876**	**1.007 - 3.494**	**0.317**	**1.982**	**0.047**
	Student - other non-health related majors	1.235	0.624 - 2.445	0.348	0.605	0.545
	Unemployed	0.806	0.406 - 1.600	0.350	-0.616	0.538
**Log-Likelihood: -484.08**	**Log-Likelihood of Null Model: -498.28**		**Log-Likelihood Ratio P value: <0.002**			

Supp. Table S1: The results of the logistic regression modelling the doctor trustworthiness as a binary variable, exploring its determinants. Rows with significant P values are bolded.

Social Media Trustworthiness – Binary Logistic Regression (LR)						
**Model Terms**		e^{β_i}	**95% CI**	**SE**	**z-Statistic**	**P value**
Intercept (β_0)		0.746	0.246 - 2.264	0.566	-0.517	0.605
**Age**	Younger than or equal to 18 years	-	-	-	-	-
	**Between 19 and 29 years, inclusive**	**0.161**	**0.085 - 0.305**	**0.326**	**-5.598**	**<0.0005**
	**Between 30 and 39 years, inclusive**	**0.333**	**0.126 - 0.876**	**0.494**	**-2.227**	**0.026**
	40 years or older	0.402	0.150 - 1.076	0.502	-1.814	0.070
**Nationality**	Non-Arab	-	-	-	-	-
	**Other Arab**	**0.448**	**0.267 - 0.749**	**0.263**	**-3.058**	**0.002**
	UAE National	0.718	0.410 - 1.257	0.286	-1.159	0.247
**Marital Status**	Unmarried	-	-	-	-	-
	Married	0.758	0.384 - 1.495	0.347	-0.800	0.423
**Sex**	Male	-	-	-	-	-
	Female	0.603	0.363 - 1.000	0.258	-1.958	0.050
**Occupation**	Healthcare (nurses, doctors, dentists, pharmacists, healthcare administration, etc.)	-	-	-	-	-
	Housewife	1.496	0.515 - 4.341	0.544	0.741	0.459
	Non-healthcare	1.659	0.715 - 3.854	0.430	1.178	0.239
	Student - health-related majors	1.133	0.464 - 2.765	0.455	0.274	0.784
	Student - other non-health related majors	0.703	0.261 - 1.895	0.506	-0.697	0.486
	Unemployed	0.756	0.241 - 2.370	0.583	-0.479	0.632
**Log-Likelihood: -342.95**	**Log-Likelihood of Null Model: -375.00**		**Log-Likelihood Ratio P value: <0.0005**			

Supp. Table S2: The results of the logistic regression modelling social media trustworthiness as a binary variable, exploring its determinants. Rows with significant P values are bolded.

Internet Trustworthiness – Binary Logistic Regression (LR)						
**Model Terms**		**e^{β_i}**	**95% CI**	**SE**	**z-Statistic**	**P value**
Intercept (β_0)		1.059	0.484 - 2.316	0.399	0.143	0.886
**Age**	Younger than or equal to 18 years	-	-	-	-	-
	**Between 19 and 29 years, inclusive**	**0.584**	**0.364 - 0.937**	**0.241**	**-2.230**	**0.026**
	Between 30 and 39 years, inclusive	0.909	0.485 - 1.704	0.320	-0.298	0.766
	40 years or older	0.646	0.349 - 1.195	0.314	-1.391	0.164
**Nationality**	Non-Arab	-	-	-	-	-
	Other Arab	0.993	0.686 - 1.436	0.189	-0.040	0.968
	UAE National	0.777	0.512 - 1.178	0.212	-1.186	0.236
**Health Literacy**	Limited Reading Ability	-	-	-	-	-
	**Normal Reading Ability**	**1.443**	**1.066 - 1.952**	**0.155**	**2.372**	**0.018**
**Sex**	Male	-	-	-	-	-
	**Female**	**0.691**	**0.503 - 0.950**	**0.163**	**-2.275**	**0.023**
**Occupation**	Healthcare (nurses, doctors, dentists, pharmacists, healthcare administration, etc.)	-	-	-	-	-
	Housewife	0.550	0.288 - 1.053	0.331	-1.804	0.071
	Non-healthcare	0.714	0.440 - 1.160	0.248	-1.363	0.173
	Student - health-related majors	0.707	0.440 - 1.137	0.243	-1.431	0.152
	**Student - other non-health related majors**	**0.385**	**0.212 - 0.699**	**0.305**	**-3.134**	**0.002**
	**Unemployed**	**0.496**	**0.261 - 0.945**	**0.328**	**-2.135**	**0.033**
**Log-Likelihood: -646.92**	**Log-Likelihood of Null Model: -669.90**		**Log-Likelihood Ratio P value: <0.0005**			

Supp. Table S3: The results of the logistic regression modelling internet trustworthiness as a binary variable, exploring its determinants. Rows with significant P values are bolded.
